# Comparison of the Intrinsic Foot Muscle Activities between Therapeutic and Three-Dimensional Foot-Ankle Exercises in Healthy Adults: An Explanatory Study

**DOI:** 10.3390/ijerph17197189

**Published:** 2020-10-01

**Authors:** Du-Jin Park, Young-In Hwang

**Affiliations:** 1 Department of Industrial Health, College of Health Sciences, Catholic University of Pusan, Busan 46252, Korea; djpark35@cup.ac.kr; 2 Department of Physical Therapy, College of Life and Health Science, Hoseo University, Asan 31499, Korea

**Keywords:** foot–ankle exercise, intrinsic foot muscle, proprioceptive neuromuscular facilitation, short foot, toe spread out

## Abstract

Background: In recent years, a three-dimensional ankle exercise has been proposed as a practice for strengthening the intrinsic foot muscles, however this topic still requires further research. This study aimed to compare the activities of the intrinsic muscles in healthy participants during 3D foot–ankle exercises, namely, short foot (SF), and toe spread out (TSO). Methods: Prior to the experiment, 16 healthy adults were trained on how to perform SF, TSO, and 3D foot–ankle exercises for an hour. Once all participants passed the foot–ankle exercise performance test, we randomly measured the activity of the intrinsic foot muscles using electromyography while the patients were performing foot–ankle exercises. Results: The abductor hallucis (AbH), extensor hallucis longus (EHL), and flexor hallucis brevis (FHB) activities showed significant differences among the exercises for intrinsic foot muscle strengthening (*p* < 0.01). Additionally, the AbH/AdH (adductor hallucis) ratio showed significant differences among the exercises for strengthening the intrinsic foot muscles (*p* < 0.01). Conclusions: Our results showed that the 3D extension exercise is as effective as the therapeutic exercise in terms of the AbH and FHB activities, and the AbH/AdH ratio. On the contrary, the 3D flexion exercise showed superiority in terms of the EHL activity.

## 1. Introduction

The foot is a complex structure designed to perform appropriate functions given various postures and tasks [1,2]. The functions of this complex structure are maintained via the interaction between the passive, active, and neural subsystems [2]. The passive subsystem consists of the bones, ligaments, and joint capsules [2]. The plantar fascia and spring ligament, its other components, are basic structures that prevent the medial longitudinal arch (MLA) from height decreases [3]. The active subsystem consists of the intrinsic and extrinsic muscles [2], with increases in strength leading to an effective recovery of the MLA height [4]. The neural subsystem consists of sensory receptors within active and passive structures [2]. In particular, plantar sensation serves as an essential role in walking and balancing [5,6].

The intrinsic muscles of the foot provide afferent information and a stable base of support for balance. When accommodating sudden changes in the supporting surface, they change the shape of the foot according to the loading [7,8]. Moreover, their short moment arm contributes to enhancing the stability of the primary arch and provides a dynamic stability to the MLA against external forces working instantaneously on the arch while walking [2].

Diseases such as plantar fasciopathy [9], hallux valgus (HV) [10], pes planus [11], lateral ankle sprains, and chronic ankle instability [12] are related to the weakness and dysfunction of the intrinsic muscles of the foot. Furthermore, in the elderly, the weakness of the intrinsic muscles can increase the risk of falling [13]. This is because the abductor hallucis (AbH), a typical intrinsic muscle, is a type II muscle fibre which is known to develop muscular atrophy and gradually decrease in the cross-section with ageing [14]. The focus during the rehabilitation phase of such foot-related diseases is strengthening the intrinsic muscle and recovering its functions. However, despite frequent clinical applications of exercises that strengthen the intrinsic muscle in foot-related diseases, there is still a gap in existing literature with respect to studies that analyse activities of the intrinsic muscle during strengthening exercises [15].

The two major exercises for strengthening the intrinsic muscles are short foot (SF) and toe spread out (TSO). Although SF is an effective exercise for height recovery of the MLA and for enhancing balance [8], TSO has been proven effective in increasing the thickness of the AbH by inducing abduction movements of the first metatarsophalangeal joint [16]. Recently, a three-dimensional (3D) ankle exercise has been proposed as a method for strengthening the intrinsic and extrinsic foot muscles. It utilises only 3D ankle movements from proprioceptive neuromuscular facilitation (PNF) lower limb extremity patterns and is recommended as an exercise [17]. Moreover, an intervention that included this exercise produced positive results in people with pes planus and lateral ankle sprains. Lee and Park (2020) reported that the 3D foot–ankle exercise using PNF improved the foot function and muscle strength of an obese individual with plantar fasciitis [17]. However, there needs to be further research on the 3D foot–ankle exercise; in particular, it is necessary to investigate its objective effects compared to those of the verified exercise for strengthening the intrinsic muscles. Thus, this study aimed to compare and analyse the activities of the intrinsic muscles in healthy participants while they perform 3D foot–ankle exercises, SF, and TSO.

## 2. Materials and Methods

### 2.1. Participants

**Figure 1:**
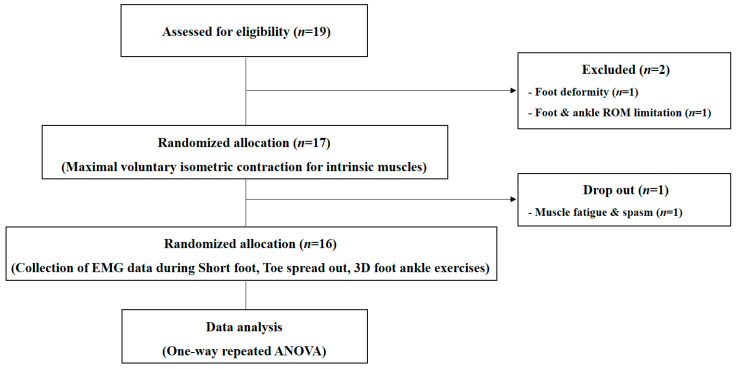
Diagram of the study design. ROM: range of motion; EMG: electromyogram; 3D: 3-dimensional; ANOVA: analysis of variance.

Nineteen participants were required for the present study design in order to achieve 80% power, a 0.25 effect size (η2), and an alpha level of 0.05. A total of 19 healthy male and female adults, who fully understood the purpose of the study and gave consent to participate in the study, were selected as participants. The inclusion criteria were as follows: no experience in foot–ankle exercises, no ankle injury and pain within the last 3 months, and aged between 20 and 30 years and residing in the local community. The participants who satisfied the following criteria but were excluded from the study either had a deformity or a disease in the foot, and were unable to perform the exercises, or had a neurological disorder, and had pain around the foot or the ankle. The descriptive statistics for the final selected participants are shown in Table 1. The entire study procedure was approved by the Catholic University of Pusan Institutional Review Board (CUPIRB-2020-017). The diagram of the study design is shown in Figure 1.

### 2.2. Measurement of Intrinsic Foot Muscles

**Figure 2:**
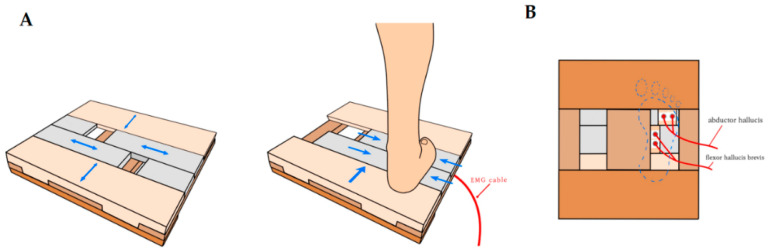
Developed EMG foot plate: (**A**) top view, (**B**) bottom view.

This study used a surface electromyogram (EMG; TeleMyo 2400T, Noraxon Inc., Scottsdale, AZ, USA) to measure the activity of the intrinsic foot muscles. The surface EMG was sampled at 1000 Hz and digitised using a 16-bit analogue to digital converter. Signals were filtered at a bandwidth of 20–400 Hz to eliminate any noise recorded during the data collection process, and a root-mean-square (RMS) window of 50 ms was used for signal smoothing. The mean of each period was calculated during the foot–ankle exercises. All electrodes were attached to the target muscles after cleaning the sites with alcohol to reduce skin impedance. To measure the activities of AbH, adductor hallucis (AdH), extensor hallucis longus (EHL), and flexor hallucis brevis (FHB), the surface electrodes were attached to the leg on the dominant side as described in a previous study [18]. For the AbH, an electrode is attached at the anterior margin of the medial malleolus, which is located in front of the imaginary line, approximately 1–2 cm posterior to the navicular tuberosity. For the AdH, it is attached at the most prominent part of the muscle belly, just proximal to the third metatarsophalangeal joint. For the EHL and FHB, the electrodes are attached at three finger widths above the bimalleolar line of the ankle, just lateral to the crest of the tibia and the most prominent muscle belly that is proximal and medial to the tendon of the flexor hallucis longus. The dominant leg was defined as the leg that is able to kick a ball farther [19]. To eliminate noise caused by floor contact, the AdH and FHB muscle activities were measured using the developed EMG plate in this study (Figure 2).

### 2.3. Maximal Voluntary Isometric Contraction (MVIC)

To normalise the activity of each muscle, we performed maximal voluntary isometric contraction (MVIC). The posture for testing each muscle was based on the posture for measuring the muscle strength [20]. All participants measured the MVIC in the supine position with the ankle exposed at the end of the table. The MVIC for each muscle was measured three times for 5 s, where the first and last seconds were discarded to compute an average value of over 3 s. In order to minimise the fatigue on intrinsic muscles due to the MVIC measurements, we performed MVIC measurements only after completing the measurements in all exercises prior to the experiment of the intrinsic foot muscles. While measuring MVIC, a 1-min break was provided as a preventive measure against muscle fatigue and spasm.

### 2.4. Foot–ankle Exercise for Strengthening of the Intrinsic Foot Muscles

**Figure 3:**
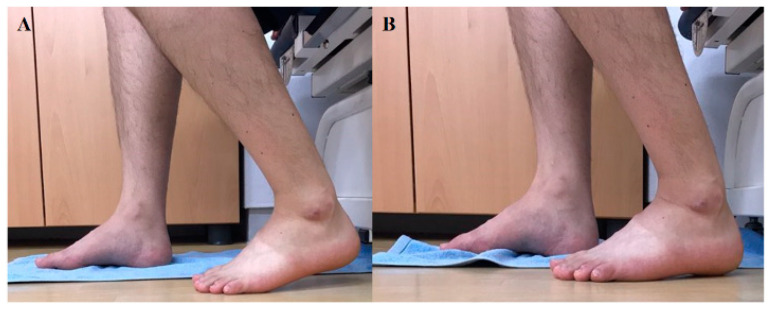
Short foot exercise: (**A**) start, (**B**) end.

**Figure 4:**
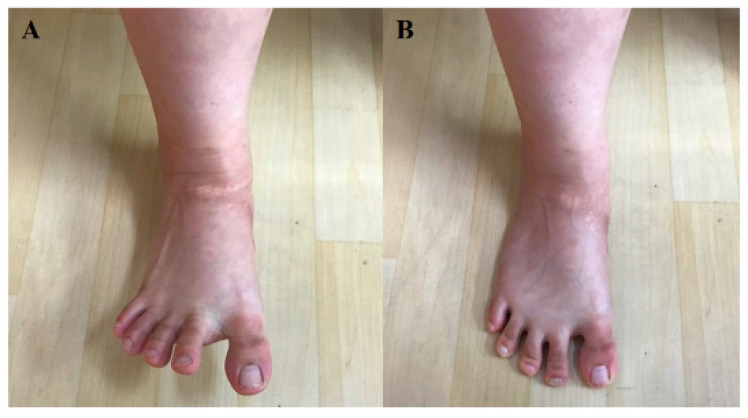
Toe spread out exercise: (**A**) start, (**B**) end.

Prior to the experiment, participants were trained on how to perform therapeutic foot–ankle and 3D foot–ankle exercises for an hour. Once all participants passed the foot–ankle exercise performance test, we randomly measured the activity of the intrinsic foot muscles while the participants were performing concentric foot–ankle exercises. As therapeutic foot–ankle exercises, SF and TSO were performed as follows: under a clinical expert’s lead, SF was passively performed in a partial weight bearing condition, during which excessive activities of the extrinsic foot muscles were reduced and activities of the intrinsic foot muscles were increased. Furthermore, passive and active movements were employed; the participants could get feedback sensation from the exercise by placing their feet on a towel on the ground (Figure 3). In TSO, while standing, the participants were asked to raise their heel and toes; to spread out all toes; to touch the ground with the heel and then the toes, in the order of the little toe, big toe, and other toes; and to hold the position [16] (Figure 4). Moreover, we used a special footrest to reduce the noise caused by the sole contacts that occurred during the two exercises.

**Figure 5:**
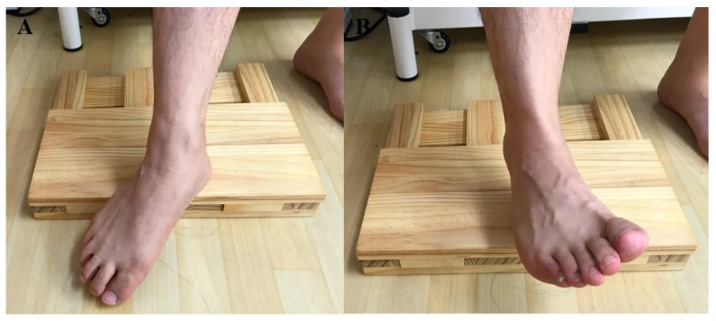
D1 patterns: (**A**) plantarflexion–pronation–eversion with toe flexion, (**B**) dorsiflexion–supination–inversion with toe extension.

**Figure 6:**
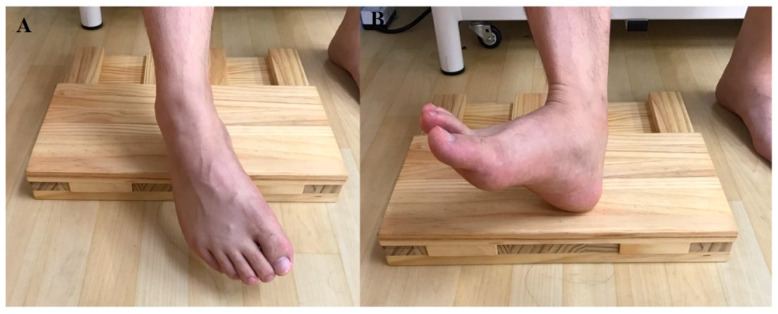
D2 patterns: (**A**) plantarflexion–supination–inversion with toe flexion, (**B**) dorsiflexion–pronation–eversion with toe extension.

The 3D foot–ankle exercise utilises 3D ankle movements in flexion and extension of Diagonals 1 (D1) and 2 (D2), which are PNF lower extremity patterns [21]. For the D1 flexion (D1F) exercise, dorsiflexion–supination–inversion with toe extension is performed from the starting posture, and then plantar flexion–pronation–eversion with toe flexion is performed (Figure 5A). The movement begins from a distal part, the toe. The D1 extension (D1E) exercise is the opposite of D1 flexion training (Figure 5B). For the D2 flexion (D2F) exercise, dorsiflexion–pronation–eversion with toe extension is performed from the starting posture, and then plantar flexion–supination–inversion with toe flexion is performed (Figure 6A). The D2 extension (D2E) training is the opposite (Figure 6B). All exercises for intrinsic foot muscle strengthening were performed in the same sitting posture three times for 5 s. The average value within 3 s, excluding the first and last seconds, was used in the analysis.

### 2.5. AbH/AdH Ratio

The AbH/AdH ratio was used to analyse the simultaneous contraction of AbH and AdH. It is an important index for evaluating the deformity and functional recovery of HV [16,18]. This study used the quantification method, which divides the %MVIC value of AdH from the %MVIC value of AbH, to compute the ratio.

### 2.6. Data Analyses

To present the general characteristics of participants in this study, we used descriptive statistics. We performed a one-way repeated analysis of variance to examine the difference in the activities of the intrinsic foot muscles caused by the foot–ankle exercise. As a post-hoc test, Bonferroni’s multiple comparison test was used. For the statistical analysis, we used SPSS 25.0 for Windows software (SPSS Inc., Chicago, IL, USA) and defined a *p*-value < 0.05 as statistically significant.

## 3. Results

**Figure 7:**
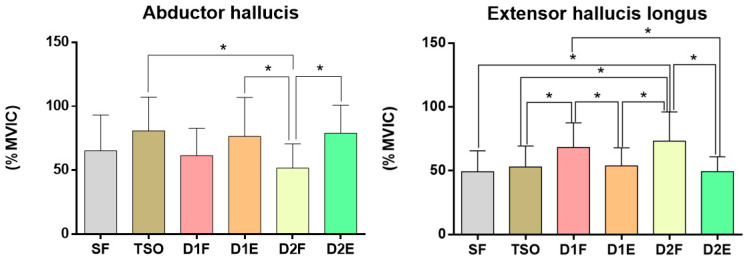
Comparison of the activation of abductor hallucis and extensor hallucis longus during foot–ankle exercises. * *p* < 0.05.

**Figure 8:**
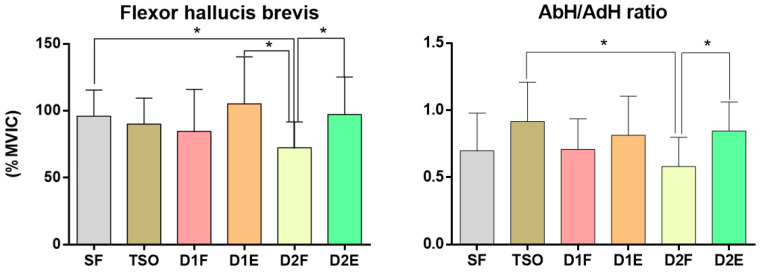
Comparison of the activation of flexor hallucis brevis and AbH/AdH ratio during foot–ankle exercises. * *p* < 0.05.

The AbH activities showed significant differences among the exercises for intrinsic foot muscle strengthening (F = 5.445, *p* < 0.01); TSO, D1E, and D2E had significantly higher AbH activities than D2F (Table 2, Figure 7). The AdH activities showed no significant difference among the exercises for intrinsic foot muscle strengthening (F = 3.491, *p* > 0.05). The EHL activities showed significant differences among the exercises (F = 11.688, *p* < 0.01); D1F had significantly higher EHL activities than TSO, D1E, and D2E (Table 2, Figure 7), and D2F had a significantly higher EHL activity than SF, TSO, D1E, and D2E (Table 2, Figure 7). FHB activities also showed significant differences among the exercises (F = 5.320, *p* < 0.01); although SF had the highest FHB activity, SF, D1E, and D2E also had a significantly higher FHB activity than D2F (Table 2, Figure 8).

The AbH/AdH ratio showed significant differences among the exercises for strengthening the intrinsic foot muscles (F = 5.331, *p* < 0.01). The ratio was significantly higher in TSO than in D2F, and D2E had a significantly higher ratio than D2F (Table 2, Figure 8).

## 4. Discussion

AbH is a typical intrinsic foot muscle that receives much attention from clinical experts owing to its various functions. In particular, the recovery of this muscle is most important for the intervention against diseases, such as HV and pes planus. For this, a basic core exercise of the foot, such as the abdominal drawing-in manoeuvre among the lumbopelvic core exercises, is used as SF [6]. However, a recent study showed that TSO is more effective in promoting AbH activity than SF [16]. Although this study did not find a significant difference between the two exercises, our finding of TSO having approximately 15% higher MVIC than SF supports the result of the previous study.

The AbH activities in this study were as follows, in the highest to lowest order: TSO, D2E, and D1E. The 3D extension exercise showed a similar level with TSO, but, in general, it was approximately 11% higher in MVIC than SF. Toe flexion is one of the typical functions of AbH [22], which also measures the strength of the intrinsic foot muscle [23], and causes muscle fatigue [24]. Moreover, a recent study argued that the reason why a detailed report on toe abduction and adduction related to the 3D extension exercise is missing in the literature originates from the traditional viewpoint, in which the intrinsic foot muscles are activated via toe flexion. It shows that the 3D extension exercise can be a sufficient alternative to TSO or SF for AbH activity.

HV patients show a disproportionate AbH/AdH ratio [18], which is used to evaluate selective AbH activities [16,18]. A closer look at studies on HV patients revealed that TSO (1.13) had a higher ratio than SF (0.59) [16]. Based on these results, we considered that an exercise with an AbH/AdH ratio near or higher than 1.0 may be appropriate for HV patients. In this study, TSO had a significantly higher ratio than D2F, with a value (0.9) relatively closer to 1.0 than any other exercises. Our result is supported by previous studies that showed a ratio of 0.9 or higher for TSO [16].

Moreover, we might have to pay attention to the ratio of 3D exercises (0.81–0.85), the next highest value to TSO. Although 3D extension exercises showed an AbH/AdH ratio above 0.81, the AdH activity was higher than those of other exercises. It may be a result of the reinforcement caused by the tibialis posterior contraction. This reinforcement generates strength by adding a new stimulus [21]. The deep attachment of the tibialis posterior is located between the flexor digitorum longus and flexor hallucis longus [25], and gives synergy to the surrounding flexor braves during plantar flexion. We considered that such a mechanism promoted the AdH activity, which has functions of toe adduction and flexion. This can be verified through the FHB activity during the 3D extension exercise in this study. Our results show that there might be limitations in applying the 3D extension to HV intervention.

On the other hand, 3D extension has an advantage of additionally activating the tibialis posterior and peroneus longus depending on exercise directions. Participants with pes planus had a reduced peroneus longus function while performing SF compared to a normal person, and they required intervention for the extrinsic foot muscles [26]. Additionally, a previous study reported that tibialis anterior muscle activity increased whereas peroneus longus muscle activity decreased in the contact phase during walking for individuals with flat feet [27]. D1E activates the peroneus longus with movements of plantar flexion and eversion and can activate the intrinsic foot muscles as well [21]. D2E contributes to the activation of tibialis posterior with movements of plantar flexion and inversion [21]. The plantar insertion of the tibialis posterior is attached to the navicular bone tubercle and the plantar sections of the medial cuneiform to provide support for the MLA [25]. McKeon et al. (2015) claimed that the balanced activities of the intrinsic and extrinsic foot muscles are important for foot stability [2]. In this perspective, 3D extension could contribute to enhancing the pes planus functions by promoting activities of the intrinsic and extrinsic foot muscles. 

In pes planus, FHB is as important as AbH. Patients with pes planus have lesser thickness and a smaller cross-sectional area of AbH and FHB than the normal person [28]. The reduced muscle thickness and smaller cross-sectional area are closely related to the reduced balance observed while standing on one foot [29]. In this study, SF had the highest FHB activity, which was significantly higher than that of the D2F exercise. Such a result supports the claim that SF is a basic foot core exercise. Furthermore, the average %MVICs of SF and D1E exercises were higher than 105%, and the average %MVIC of D2E (97%) was close to 100% as well. The causes of these findings include the crosstalk from the EMG attachment sites and the noise from the sole contacts during the exercises; however, they could also be due to a lack of sensitivity of the general posture during the MVIC measurement of the intrinsic foot muscles. Hence, research on MVIC postures that can induce independent FHB contractions is required. 

In EHL, the 3D flexion exercise induced a significantly higher activity than the other exercises, which is due to the extension of the big toe, dorsiflexion, medial and lateral eversion, and EHL function that increases the elongation of plantar aponeurosis [30,31]. Although it is still unclear whether this muscle is closely related to forefoot deformities, such as HV [32], the tendon displacement by EHL among the surgical intervention methods for HV patients has proven its effect [33]. However, there is a gap in research that pays attention to the EHL activity as a conservative approach for HV. Given that AbH loses its anti-valgus function in HV patients, AdH plays a dominant role in allowing the medial and plantar migration to achieve a more inferior position, and it works mainly as the flexor muscles for the toes [34]. It supports the findings of previous studies, which reported that HV patients experience difficulty in independently performing toe abduction on the transverse plane [16,18]. Furthermore, such a mechanism causes the metatarsal head to move medially and the moment arm of the flexor and extensor muscles to move laterally. Consequently, EHL also applies value force on the big toe [33]. Such deformity renders the precise performance of exercise difficult due to the bowstring effect [18]. Hence, a conservative exercise that can bring changes to the valgus mechanism is required. The exercise developed in previous studies, wherein the big toe is abducted and adducted at the same time, must have originated from such a perspective.

There are three limitations in this study. First, given that we used surface EMG to monitor the muscle activity of the intrinsic foot muscles, the pitfall of crosstalk suggested in previous studies could not be avoided [33]. Moreover, there were difficulties in removing the noise caused by sole contacts while the patients were performing the exercises. Further research on the methods of investigating the activities of intrinsic foot muscles, including measurement methods that combine surface EMG and ultrasonography and methods that can be applied to the abdominal muscles, is needed [34]. Second, owing to the small population size and the fact that the study was conducted on normal people, our results are limited in terms of generalising them to patients with pes planus or HV. In the future, we hope to conduct random clinical trials that apply 3D foot–ankle exercises to patients with pes planus or HV. Finally, a measurement instrument was not used to evaluate the possible changes that the participant may experience during and after performing the exercises.

## 5. Conclusions

In conclusion, our results showed that the 3D extension exercise is as effective as the therapeutic exercise in terms of the AbH and FHB activities and the AbH/AdH ratio. On the contrary, the 3D flexion exercise showed superiority in terms of the EHL activity.

## Figures and Tables

**Table 1:** Descriptive statistics of the study participants (*n* = 16).

Variable	Mean ± SD ^1^
Age (years)	27.44 ± 2.58
Height (cm)	166.19 ± 6.80
Weight (kg)	59.75 ± 9.54
BMI ^2^ (kg/m^2^)	21.57 ± 2.62
Sex	Male 8 (50%), female 8 (50%)
Dominant foot	Right = 13 (81.2%), left = 3 (18.8%)

^1^ SD: standard deviation; ^2^ BMI: body mass index.

**Table 2:** Descriptive statistics of normalised electromyogram data of the four muscles and abductor hallucis/adductor hallucis ratio during foot strengthening exercises (*n* = 16)**.**

Variable	SF ^5^	TSO ^6^	D1F ^7^	D1E ^8^	D2F ^9^	D2E ^10^
AbH ^1^ (%MVIC) *	65.06 ± 28.01	80.84 ± 26.33	61.41 ± 21.33	76.48 ± 30.40	51.00 ± 17.20	78.97 ± 22.00
AdH ^2^ (%MVIC)	92.36 ± 8.16	88.10 ± 7.66	86.18 ± 7.40	93.37 ± 10.49	88.95 ± 5.84	92.96 ± 8.17
EHL ^3^ (%MVIC) *	49.16 ± 16.44	52.92 ± 19.37	68.14 ± 19.37	53.75 ± 14.22	73.19 ± 22.83	49.35 ± 11.56
FHB ^4^ (%MVIC) *	95.96 ± 19.54	90.09 ± 19.40	84.58 ± 31.35	105.14 ± 35.15	72.28 ± 19.35	97.23 ± 27.97
AbH/AdH ratio *	0.70 ± 0.28	0.92 ± 0.29	0.71 ± 0.23	0.81 ± 0.29	0.58 ± 0.22	0.85 ± 0.22

^1^ AbH: abductor hallucis, ^2^ AdH: adductor hallucis, ^3^ EHL: extensor hallucis longus, ^4^ FHB: flexor hallucis brevis, ^5^ SF: short foot exercise, ^6^ TSO: toe spread out, ^7^ D1F: diagonal 1 flexion pattern, ^8^ D1E: diagonal 1 extension pattern, ^9^ D2F: diagonal 2 flexion pattern, ^10^ D2E: diagonal 2 extension pattern, * *p* < 0.05.

## References

[1] Saltzman C.L., Nawoczenski D.A. (1995). Complexities of foot architecture as a base of support. J. Orthop. Sports Phys. Ther..

[2] McKeon P.O., Hertel J., Bramble D., Davis I. (2015). The foot core system: A new paradigm for understanding intrinsic foot muscle function. Br. J. Sports Med..

[3] Iaquinto J.M., Wayne J.S. (2010). Computational model of the lower leg and foot/ankle complex: Application to arch stability. J. Biomech. Eng..

[4] Snyder K.R., Earl J.E., O’Connor K.M., Ebersole K.T. (2009). Resistance training is accompanied by increases in hip strength and changes in lower extremity biomechanics during running. Clin. Biomech..

[5] Eils E., Nolte S., Tewes M., Thorwesten L., Völker K., Rosenbaum D. (2002). Modified pressure distribution patterns in walking following reduction of plantar sensation. J. Biomech..

[6] McKeon P.O., Hertel J. (2007). Diminished plantar cutaneous sensation and postural control. Perceptual Mot. Skills.

[7] Kelly L.A., Cresswell A.G., Racinais S., Whiteley R., Lichtwark G. (2014). Intrinsic foot muscles have the capacity to control deformation of the longitudinal arch. J. R. Soc. Interface.

[8] Mulligan E.P., Cook P.G. (2013). Effect of plantar intrinsic muscle training on medial longitudinal arch morphology and dynamic function. Man. Ther..

[9] Chang R., Kent-Braun J.A., Hamill J. (2012). Use of MRI for volume estimation of tibialis posterior and plantar intrinsic foot muscles in healthy and chronic plantar fasciitis limbs. Clin. Biomech..

[10] Stewart S., Ellis R., Heath M., Rome K. (2013). Ultrasonic evaluation of the abductor hallucis muscle in hallux valgus: A cross-sectional observational study. BMC Musculoskeletal Disord..

[11] Angin S., Mickle K.J., Nester C.J. (2018). Contributions of foot muscles and plantar fascia morphology to foot posture. Gait Posture.

[12] Feger M.A., Snell S., Handsfield G.G., Blemker S.S., Wombacher E., Fry R., Hart J.M., Saliba S.A., Park J.S., Hertel J. (2016). Diminished foot and ankle muscle volumes in young adults with chronic ankle instability. Orthop. J. Sports Med..

[13] Mickle K.J., Munro B.J., Lord S.R., Menz H.B., Steele J.R. (2009). Toe weakness and deformity increase the risk of falls in older people. Clin. Biomech..

[14] Aiyer A., Stewart S., Rome K. (2015). The effect of age on muscle characteristics of the abductor hallucis in people with hallux valgus: A cross-sectional observational study. J. Foot Ankle Res..

[15] Gooding T.M., Feger M.A., Hart J.M., Hertel J. (2016). Intrinsic foot muscle activation during specific exercises: A T2 time magnetic resonance imaging study. J. Athl. Train..

[16] Kim M.H., Kwon O.Y., Kim S.H., Jung D.Y. (2013). Comparison of muscle activities of abductor hallucis and adductor hallucis between the short foot and toe-spread-out exercises in subjects with mild hallux valgus. J. Back Musculoskeletal Rehabil..

[17] Lee K.S., Park D.J. (2020). Three-dimensional ankle exercise with combined isotonic technique for an obese subject with plantar fasciitis: A case study. Medicina.

[18] Arinci Incel N., Genç H., Erdem H.R., Yorgancioglu Z.R. (2003). Muscle imbalance in hallux valgus: An electromyographic study. Am. J. Phys. Med. Rehabil..

[19] Letafatkar A., Rajabi R., Minoonejad H., Rabiei P. (2019). Efficacy of perturbation-enhanced neuromuscular training on hamstring and quadriceps onset time, activation and knee flexion during a tuck-jump task. Int. J. Sports Phys. Ther..

[20] Hislop H., Avers D., Brown M. (2013). Daniels and Worthingham’s Muscle Testing.

[21] Adler S.S., Beckers D., Buck M. (2014). PNF in Practice: An Illustrated Guide.

[22] Tosovic D., Ghebremedhin E., Glen C., Gorelick M., Mark Brown J. (2012). The architecture and contraction time of intrinsic foot muscles. J. Electromyogr. Kinesiol..

[23] Soysa A., Hiller C., Refshauge K., Burns J. (2012). Importance and challenges of measuring intrinsic foot muscle strength. J. Foot Ankle Res..

[24] Headlee D.L., Leonard J.L., Hart J.M., Ingersoll C.D., Hertel J. (2008). Fatigue of the plantar intrinsic foot muscles increases navicular drop. J. Electromyogr. Kinesiol..

[25] Corcoran N.M., Varacallo M. (2020). Anatomy, Bony Pelvis and Lower Limb, Tibialis Posterior Muscle.

[26] Park D.J., Park S.Y. (2018). Comparison of subjects with and without pes planus during short foot exercises by measuring muscular activities of ankle and navicular drop height. J. Korean Soc. Phys. Med..

[27] Murley G.S., Menz H.B., Landorf K.B. (2009). Foot posture influences the electromyographic activity of selected lower limb muscles during gait. J. Foot Ankle Res..

[28] Angin S., Crofts G., Mickle K.J., Nester C.J. (2014). Ultrasound evaluation of foot muscles and plantar fascia in pes planus. Gait Posture.

[29] Taş S., Ünlüer N.Ö., Çetin A. (2020). Thickness, cross-sectional area, and stiffness of intrinsic foot muscles affect performance in single-leg stance balance tests in healthy sedentary young females. J. Biomech..

[30] Moore K., Dalley A. (2006). Lower Limb. Clinically Oriented Anatomy.

[31] Natsis K., Konstantinidis G.A., Symeonidis P.D., Totlis T., Anastasopoulos N., Stavrou P. (2017). The accessory tendon of extensor hallucis longus muscle and its correlation to hallux valgus deformity: A cadaveric study. Surg. Radiol. Anat..

[32] Zhang F.Q., Wang H.J., Zhang Q., Liu Y.L., Zhang Y.Z. (2010). Hallux valgus deformity treated with the extensor hallucis longus tendon transfer by dynamic correction. Chin. Med. J..

[33] Solomonow M., Baratta R., Bernardi M., Zhou B., Lu Y., Zhu M., Acierno S. (1994). Surface and wire EMG crosstalk in neighbouring muscles. J. Electromyogr. Kinesiol..

[34] Yang K.H., Park D.J. (2014). Reliability of ultrasound in combination with surface electromyogram for evaluating the activity of abdominal muscles in individuals with and without low back pain. J. Exercise Rehabil..

